# CH_3_NH_3_PbI_3_/Au/Mg_0.2_Zn_0.8_O Heterojunction Self-Powered Photodetectors with Suppressed Dark Current and Enhanced Detectivity

**DOI:** 10.3390/ma16124330

**Published:** 2023-06-12

**Authors:** Meijiao Wang, Man Zhao, Dayong Jiang

**Affiliations:** 1 School of Materials Science and Engineering, Changchun University of Science and Technology, Changchun 130022, China; 2019200119@mails.cust.edu.cn; 2 School of Optoelectronic Engineering, Changchun College of Electronic Technology, Changchun 130022, China; 3 Engineering Research Center of Optoelectronic Functional Materials, Ministry of Education, Changchun 130022, China

**Keywords:** CH_3_NH_3_PbI_3_ photodetectors, heterojunction, self-powered photodetectors, Mg_0.2_Zn_0.8_O

## Abstract

Interface engineering of the hole transport layer in CH_3_NH_3_PbI_3_ photodetectors has resulted in significantly increased carrier accumulation and dark current as well as energy band mismatch, thus achieving the goal of high-power conversion efficiency. However, the reported heterojunction perovskite photodetectors exhibit high dark currents and low responsivities. Herein, heterojunction self-powered photodetectors, composed of p-type CH_3_NH_3_PbI_3_ and n-type Mg_0.2_Zn_0.8_O, are prepared through the spin coating and magnetron sputtering. The obtained heterojunctions exhibit a high responsivity of 0.58 A/W, and the EQE of the CH_3_NH_3_PbI_3_/Au/Mg_0.2_Zn_0.8_O heterojunction self-powered photodetectors is 10.23 times that of the CH_3_NH_3_PbI_3_/Au photodetectors and 84.51 times that of the Mg_0.2_ZnO_0.8_/Au photodetectors. The built-in electric field of the p-n heterojunction significantly suppresses the dark current and improves the responsivity. Remarkably, in the self-supply voltage detection mode, the heterojunction achieves a high responsivity of up to 1.1 mA/W. The dark current of the CH_3_NH_3_PbI_3_/Au/Mg_0.2_Zn_0.8_O heterojunction self-powered photodetectors is less than 1.4 × 10^−1^ pA at 0 V, which is more than 10 times lower than that of the CH_3_NH_3_PbI_3_ photodetectors. The best value of the detectivity is as high as 4.7 × 10^12^ Jones. Furthermore, the heterojunction self-powered photodetectors exhibit a uniform photodetection response over a wide spectral range from 200 to 850 nm. This work provides guidance for achieving a low dark current and high detectivity for perovskite photodetectors.

## 1. Introduction

With the rapid development of two-dimensional (2D) materials, such as graphene, transition metal dichlorides (TMDs), and black phosphorus, 2D perovskites have emerged, which combine the excellent properties of 2D materials and perovskites, namely the good solution processability, molecular-scale self-assembly, and film formation of the former alongside the direct tunable band gap, high carrier mobility, and high absorption coefficient of the latter [1,2,3,4,5,6,7]. Among these, CH_3_NH_3_PbI_3_ possesses excellent absorption and has been applied to photodetectors (PDs); in particular, self-powered PDs (SPPDs) have drawn considerable research interest [8]. SPPDs can detect light without the need for any power supply; furthermore, they can be miniaturized and integrated into nanodevices with remote wireless control. However, due to the complex production process, existing perovskite SPPDs show a high dark current and a low detection rate, which greatly limits their wide application. For example, a common approach to realizing perovskite SPPDs is to construct p-i-n with vertical multilayer structures, which require complex and rigorous processes [9,10,11,12]. They are important candidates in the field of renewable photovoltaics and photoelectric devices [13,14,15,16]. Since MAPbI_3_/TiO_2_ PDs were reported in 2014, various PDs have been successfully fabricated using MAPbI_3_. However, it has been shown that perovskite SPPDs exhibit high dark currents and low responsivities [17]. Furthermore, perovskite heterojunction PDs are being quickly developed as well [18,19,20,21]. The built-in electric field formed at the junction interface can act as a driving force to separate the photogenerated electron-hole (e-h) pairs in the depletion region, drive the transport of the separated photogenerated carriers, and then generate the photocurrent in the PDs without an external power supply. Herein, heterojunction SPPDs are designed.

Due to their advantages of lightweight and easy processing, perovskites have attracted more and more attention, and they have shown great application potential in various fields in recent years. In particular, wideband organic photoelectric detection (OPD) has been successfully applied in many important fields, such as astronomical exploration, remote sensing, and infrared imaging. The combination of MgZnO and the advantages of organic polymers can improve the performance of PDs, so organic PDs show fascinating characteristics. Perovskite heterojunction PDs performance can be improved by using metal oxide dense films, such as MgZnO or ZnO, which are usually used as an electron-transport layer to transport electrons and holes [22,23,24,25]. MgZnO and ZnO with wide band gaps exhibit a large ultraviolet (UV)-light absorption coefficient and high carrier mobility. It is well known that intrinsic point defects, such as oxygen vacancies and metal interstitials, have an important impact on the electronic properties of metal oxides. It has been widely accepted that the presence of oxygen vacancies in Mg_0.2_Zn_0.8_O can increase the charge density of Mg_0.2_Zn_0.8_O to form a native n-type semiconductor [26,27,28,29]. Thus, it is urgently required to fabricate PDs with a simple alternative method that can also endow them with self-powered functionality. The most common method for fabricating SPPDs relies on the E_b_ created by heterojunctions. To date, Mg_0.2_Zn_0.8_O has been rarely reported for use as an electron-transport layer in heterojunction SPPDs. Mg_0.2_Zn_0.8_O has a wide band gap and acts as the n-type layer. CH_3_NH_3_PbI_3_/Au/Mg_0.2_Zn_0.8_O heterojunction SPPDs with excellent comprehensive properties have been successfully prepared. Therefore, it is very important to develop CH_3_NH_3_PbI_3_/Au/Mg_0.2_Zn_0.8_O heterojunction SPPDs with a low dark current and high detectivity to improve their performance.

Herein, high-quality CH_3_NH_3_PbI_3_ perovskites were prepared as hole-transport layers through the one-step spin coating method and were then used in Mg_0.2_Zn_0.8_O PDs prepared by magnetron sputtering. These devices possess light responsiveness in a broad range, from UV to near-infrared (NIR), high responsivity, and low dark current. The CH_3_NH_3_PbI_3_/Au/Mg_0.2_Zn_0.8_O heterojunction SPPDs exhibit an excellent responsivity of up to 0.58 A/W. In the self-supply voltage detection mode, it achieves a high responsivity of up to 1.1 mA/W, and the dark current is less than 1.4 × 10^−1^ pA at 0 V. The best value of the detectivity is as high as 4.7 × 10^12^ Jones. This work presents a new route for designing SPPDs with a low dark current and high detectivity.

## 2. Experimental Section

### 2.1. Materials

All the reagents used in the experiments were of analytic grade and used without further purification. The conjugated polymers PbI_2_ and CH_3_NH_3_I were purchased from Xi’an Polymer Light Technology Corp., Xi’an, China.

### 2.2. PDs Preparation Process

#### 2.2.1. Preparation of the CH_3_NH_3_PbI_3_/Au PDs

Firstly, Au thin films were prepared on a 2 × 3 cm^2^ polyethylene terephthalate (PET) using radio-frequency (RF) magnetron sputtering (JZ-RF600A). Subsequently, the metal semiconductor metal (MSM) structure was realized through the following steps: gluing, exposure, development, etching, and resist removal. The electrode width and spacing were both 5 µm, and the electrode length was 500 µm. We masked the PET film with 5 μm-wide channels with polyimide tape. In the next step, using a vacuum spin coater (VTC-200), the CH_3_NH_3_PbI_3_ layer was deposited on the PET substrate. Briefly, 10 mg of CH_3_NH_3_I and 460 mg of PbI_2_ were dissolved in 1 mL of N, N-dimethylformamide (DMF) and stirred at 70 °C for 2 h to obtain a homogeneous CH_3_NH_3_PbI_3_ precursor solution. Then, the above precursor solution was spin coated on the PET substrate at an initial speed of 500 r/min for 10 s and then at a speed of 3000 r/min for 50 s. Subsequently, PET with a spin coating was heated in a vacuum oven at 70 °C for 3 min to remove the residual DMF. After cooling, the polyimide tape was removed, and the CH_3_NH_3_PbI_3_ layer was obtained upon annealing at 90 °C for 10 min. Finally, the CH_3_NH_3_PbI_3_/Au PDs were obtained.

#### 2.2.2. Preparation of the Au/Mg_0.2_Zn_0.8_O PDs

**Figure 1:**
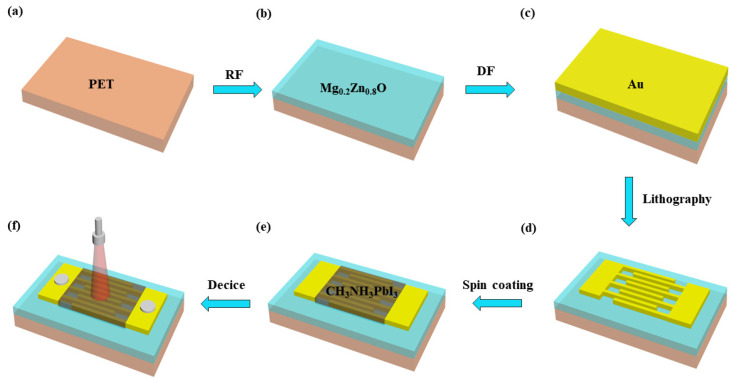
Preparation process of the CH_3_NH_3_PbI_3_/Au/Mg_0.2_Zn_0.8_O flexible PDs. (**a**) PET substrate. (**b**) Preparation of Mg_0.2_Zn_0.8_O/Au thin films. (**c**) Preparation of Au thin films. (**d**) Photolithography. (**e**) Preparation of CH_3_NH_3_PbI_3_ thin films. (**f**) Preparation of CH_3_NH_3_PbI_3_/Au/Mg_0.2_Zn_0.8_O SPPDs.

Mg_0.2_Zn_0.8_O thin films were prepared on a 2 × 3 cm^2^ PET using RF magnetron sputtering (JZ-RF600A). The vacuum chamber was initially evacuated to 5 × 10^−4^ Pa, and O_2_ and Ar were introduced into the chamber in a flow ratio of 10:40. PET was washed with acetone, absolute ethanol, and deionized water for 10 min. The Mg_0.2_Zn_0.8_O films were sputtered on the PET substrates at a total pressure of 4 Pa and an RF power of 150 W for 30 min to obtain the Mg_0.2_Zn_0.8_O thin films, as shown in Figure 1a,b.

Next, Au was RF sputtered on the Mg_0.2_Zn_0.8_O thin films to realize the MSM structure, as shown in Figure 1c,d; the process included gluing, exposure, development, etching, and resist removal. The electrode width and spacing were both 5 µm, and the electrode length was 500 µm. The obtained Au/Mg_0.2_Zn_0.8_O UV PDs are shown in Figure 1d.

#### 2.2.3. Preparation of the CH_3_NH_3_PbI_3_/Au/Mg_0.2_Zn_0.8_O Heterojunction SPPDs

Firstly, we masked the Mg_0.2_Zn_0.8_O film with 5 μm-wide channels with the polyimide tape. In the second step, using a vacuum spin coater (VTC-200), the CH_3_NH_3_PbI_3_ layer was deposited on the Mg_0.2_Zn_0.8_O thin film. 10 mg of CH_3_NH_3_I and 460 mg of PbI_2_ were dissolved in 1 mL of DMF and stirred at 70 °C for 2 h to obtain a homogeneous CH_3_NH_3_PbI_3_ precursor solution. Then, the above precursor solution was spin coated on the Mg_0.2_Zn_0.8_O thin film at an initial speed of 500 r/min for 10 s and then at a speed of 3000 r/min for 50 s. Then, a Mg_0.2_Zn_0.8_O thin film with spin coating was heated in a vacuum oven at 70 °C for 3 min to remove the residual DMF. After cooling, the polyimide tape was removed, and a CH_3_NH_3_PbI_3_ layer was obtained upon annealing at 90 °C for 10 min. Finally, the CH_3_NH_3_PbI_3_/Au/Mg_0.2_Zn_0.8_O heterojunction SPPDs were obtained, as shown in Figure 1e,f.

### 2.3. Device Characterization

The crystal structures of CH_3_NH_3_PbI_3_/Au PDs and CH_3_NH_3_PbI_3_/Au/Mg_0.2_Zn_0.8_O heterojunction SPPDs were characterized using a Rigaku Ultima VI X-ray diffractometer (XRD). The morphology was characterized by scanning electron microscopy (SEM) using a JEOL JSM-7600F microscope. The UV-visible (Vis)-NIR absorption spectra of the CH_3_NH_3_PbI_3_/Au PDs and CH_3_NH_3_PbI_3_/Au/Mg_0.2_Zn_0.8_O heterojunction SPPDs were measured using a PerkinElmer Lambda 950 spectrophotometer. The dark and photocurrent-voltage (I-V) curves of the CH_3_NH_3_PbI_3_/Au PDs and CH_3_NH_3_PbI_3_/Au/Mg_0.2_Zn_0.8_O heterojunction SPPDs were measured using an Agilent 16442A test fixture. The responsivity spectra of the CH_3_NH_3_PbI_3_/Au PDs and CH_3_NH_3_PbI_3_/Au/Mg_0.2_Zn_0.8_O heterojunction SPPDs were measured using a Zolix DR800-CUST testing system.

## 3. Results and Discussion

**Figure 2:**
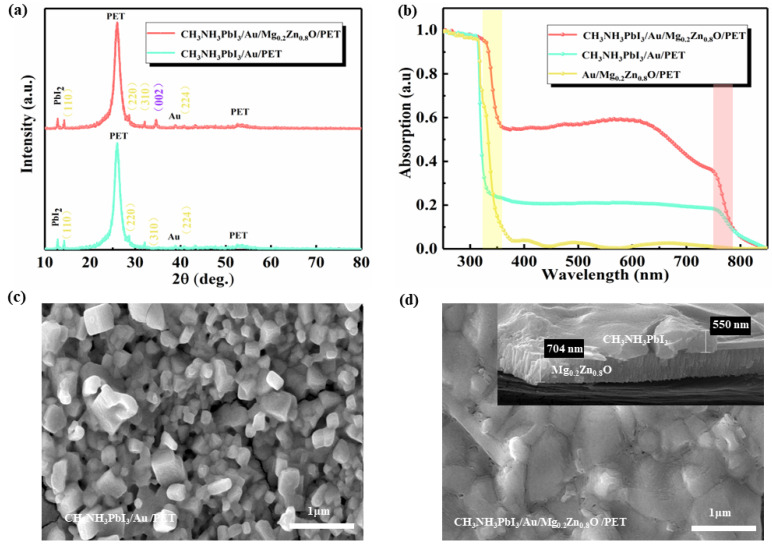
(**a**) XRD spectra of the CH_3_NH_3_PbI_3_/Au PDs and CH_3_NH_3_PbI_3_/Au/Mg_0.2_Zn_0.8_O SPPDs. (**b**) Normalized absorption spectra of the Mg_0.2_Zn_0.8_O/Au PDs, CH_3_NH_3_PbI_3_/Au PDs, and CH_3_NH_3_PbI_3_/Au/Mg_0.2_Zn_0.8_O SPPDs. (**c**,**d**) SEM spectra of the CH_3_NH_3_PbI_3_ PDs and CH_3_NH_3_PbI_3_/Au/Mg_0.2_Zn_0.8_O SPPDs.

As shown in Figure 2a, the XRD pattern of the CH_3_NH_3_PbI_3_/Au PDs exhibits diffraction peaks at 2θ = 14.1°, 28.4°, 31.9°, and 40.8°, which are associated with the (110), (220), (310), and (224) planes, respectively; the strongest diffraction peaks are (110) and (220), which shows that the materials grow preferentially along the (110) direction, which is consistent with previous studies [30,31,32]. The still-remaining diffraction peak at 12.65° suggests the level of the PbI_2_ impurity phase [33]. The XRD pattern of the CH_3_NH_3_PbI_3_/Au/Mg_0.2_Zn_0.8_O heterojunction SPPDs shows a peak at 34.51°, which corresponds to the (002) planes of Mg_0.2_Zn_0.8_O [34,35]. The other peaks are coincident with those of CH_3_NH_3_PbI_3_, indicating the diffraction peak of Mg_0.2_Zn_0.8_O film. The Mg_0.2_Zn_0.8_O thin film has no effect on the crystallinity of the CH_3_NH_3_PbI_3_ layer.

Figure 2b shows the normalized absorption spectra of the Mg_0.2_Zn_0.8_O/Au PDs, CH_3_NH_3_PbI_3_/Au PDs, and CH_3_NH_3_PbI_3_/Au/Mg_0.2_Zn_0.8_O heterojunction SPPDs. For the Mg_0.2_Zn_0.8_O/Au PDs, there is an absorption peak at a wavelength of 330 nm. A broad absorption band from 330 to 780 nm is observed for the CH_3_NH_3_PbI_3_/Au PDs. It is worth noting that the absorption of the CH_3_NH_3_PbI_3_/Au/Mg_0.2_Zn_0.8_O heterojunction SPPDs is in the range from 330 to 780 nm, and the absorption performance is better than that of the CH_3_NH_3_PbI_3_/Au PDs. Overall, the excellent light absorption properties make CH_3_NH_3_PbI_3_/Au/Mg_0.2_Zn_0.8_O heterojunction SPPDs promising candidates for high-performance PDs. It can be seen from Figure 2c,d that the cuboid-shaped CH_3_NH_3_PbI_3_ grains are uniformly distributed on the substrate. Through the double-layer film of Mg_0.2_Zn_0.8_O and CH_3_NH_3_PbI_3_, it can be seen that the upper layer of the CH_3_NH_3_PbI_3_ polycrystalline film is more dense; at the same time, the crystal grains of CH_3_NH_3_PbI_3_ can be enlarged. Larger CH_3_NH_3_PbI_3_ grains mean fewer perovskite grain boundaries. This result suggests that the Mg_0.2_Zn_0.8_O film can passivate the surface defects of the perovskite and that a uniform and flat CH_3_NH_3_PbI_3_ layer is formed.

**Figure 3:**
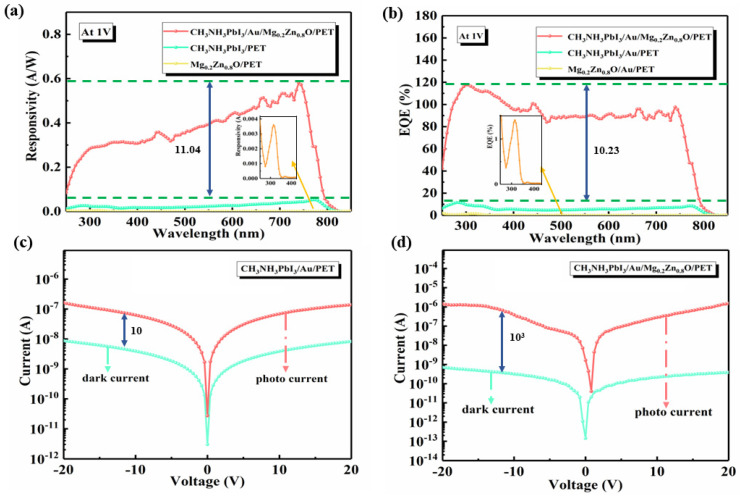
(**a**) Responsivity spectra of the CH_3_NH_3_PbI_3_/Au PDs, Mg_0.2_Zn_0.8_O PDs, and CH_3_NH_3_PbI_3_/Au/Mg_0.2_Zn_0.8_O heterojunction SPPDs under 1 V. (**b**) EQE of the CH_3_NH_3_PbI_3_/Au PDs, Mg_0.2_Zn_0.8_O PDs, and CH_3_NH_3_PbI_3_/Au/Mg_0.2_Zn_0.8_O heterojunction SPPDs under 1 V. (**c**,**d**) I-V curves of the CH_3_NH_3_PbI_3_/Au PDs and CH_3_NH_3_PbI_3_/Au/Mg_0.2_Zn_0.8_O heterojunction SPPDs under dark and light conditions.

Figure 3a shows the responsivity (R) spectra of the CH_3_NH_3_PbI_3_/Au/Mg_0.2_Zn_0.8_O heterojunction SPPDs in the UV-Vis-NIR range. It can be seen that in the range of 250–850 nm, the R value is significantly higher than that of the Mg_0.2_Zn_0.8_O/Au PDs or CH_3_NH_3_PbI_3_/Au PDs under 1 V. For the CH_3_NH_3_PbI_3_/Au/Mg_0.2_Zn_0.8_O heterojunction SPPDs, the highest R value is up to 0.58 A/W, which is 11.09 times higher than that of the best CH_3_NH_3_PbI_3_/Au PDs and is 161 times higher than that of the best Mg_0.2_Zn_0.8_O/Au PDs. It can be seen that the CH_3_NH_3_PbI_3_/Au/Mg_0.2_Zn_0.8_O PDs have a higher R than the Mg_0.2_Zn_0.8_O/Au PDs and the CH_3_NH_3_PbI_3_/Au PDs alone.

Figure 3b shows a comparison of the external quantum efficiency (EQE) measured under 1 V. The EQE curve is similar to the absorbance curve of the CH_3_NH_3_PbI_3_/Au PDs. The EQE is one of the most important performance parameters of perovskite PDs; the EQE represents the number of electron-hole pairs generated for a single incident photon. The EQE of the two groups of devices was calculated using formula (1). In the case of a certain bias, the EQE of the CH_3_NH_3_PbI_3_/Au/Mg_0.2_Zn_0.8_O heterojunction SPPDs is 10.23 times that of the CH_3_NH_3_PbI_3_/Au PDs and 84.51 times that of the Mg_0.2_ZnO_0.8_/Au PDs. The EQE is defined as:

(1) $$EQE\left(\lambda \right)=\frac{R\times h\times c}{q\times \lambda }$$

The I-V curves of the CH_3_NH_3_PbI_3_/Au PDs and CH_3_NH_3_PbI_3_/Au/Mg_0.2_Zn_0.8_O heterojunction SPPDs under dark and light conditions are shown in Figure 3c,d. The light-dark current ratio of the CH_3_NH_3_PbI_3_/Au/Mg_0.2_Zn_0.8_O heterojunction SPPDs is 100 times that of the CH_3_NH_3_PbI_3_/Au PDs. Additionally, the nonlinear I-V curves indicate that Schottky metal-semiconductor contacts were formed. By comparing Figure 3c,d, it is found that this is mainly attributed to the fact that the heterojunction inhibits the rise of the dark current. From Figure 3d, it can be seen that the dark current of the CH_3_NH_3_PbI_3_/Au/Mg_0.2_Zn_0.8_O heterojunction SPPDs is very low, and the light-dark current ratio is about 10^3^. The dark current of the CH_3_NH_3_PbI_3_/Au/Mg_0.2_Zn_0.8_O heterojunction SPPDs is less than 1.4 × 10^−1^ pA at 0 V, which is more than 10 times less than that of the CH_3_NH_3_PbI_3_ PDs. Furthermore, the dark current is very low, which can effectively reduce the lowest detectable optical power and enhance the capability of detecting weak light [36]. When the CH_3_NH_3_PbI_3_ thin film is spin coated on Mg_0.2_Zn_0.8_O, the dark current of the device decreases, the photocurrent increases, and the photoresponsivity gain is enhanced for the heterojunction. There are few carrier-donating defects in the bilayer, and the interfacial charge transfer reduces the carrier concentration in the dissipative region. The higher the light current-dark current ratio, the better the device detection performance. At the same time, the recombination of the electron pairs in the Mg_0.2_Zn_0.8_O thin film is reduced in the gap between the CH_3_NH_3_PbI_3_ grains, which is beneficial for the hole separation and transport in the CH_3_NH_3_PbI_3_/Au/Mg_0.2_Zn_0.8_O heterojunction thin film; this enables the realization of a high response and a very low dark current.

**Figure 4:**
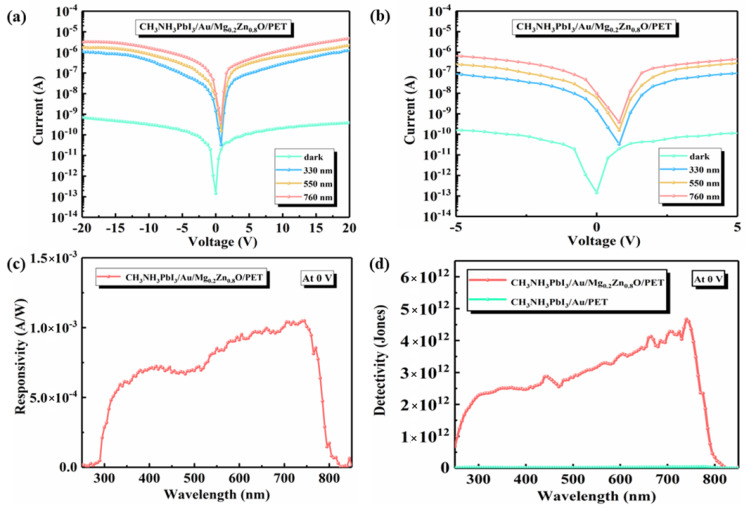
(**a**,**b**) I-V curves of the CH_3_NH_3_PbI_3_/Au/Mg_0.2_Zn_0.8_O heterojunction PDs for different wavelengths. (**c**) The responsiveness curves of the CH_3_NH_3_PbI_3_/Au/Mg_0.2_Z_n0.8_O heterojunction SPPDs at 0 V. (**d**) D* values of the CH_3_NH_3_PbI_3_/Au PDs and CH_3_NH_3_PbI_3_/Au/Mg_0.2_Zn0_.8_O heterojunction SPPDs at 0 V.

Figure 4a,b show the I-V curves for different wavelengths (namely 330, 550, and 760 nm). Clearly, the photocurrent density increases gradually with the incident light wavelength. The main reason is that the dark current is ultimately limited by the recombination current, which is an inherent property of semiconductor materials and heterojunctions. The built-in electric field of the CH_3_NH_3_PbI_3_/Au/Mg_0.2_Zn_0.8_O heterojunction with the increased Fermi level of Mg_0.2_Zn_0.8_O provides a strong driving force to separate and transfer the photogenerated carriers, and the depletion layer becomes wider, which decreases the recombination of the carriers and then reduces the dark current. This endows the heterojunction devices with a high photoresponse performance under external bias. The high detection rate of the perovskite PDs is mainly due to their very low dark current under reverse bias. Such a small dark current explains the reason for its high photodetection and also shows that perovskite-based PDs can exhibit very good detection capability. Moreover, the device shows an apparent photovoltaic behavior under illumination, and the offset voltage is 0 V, which exhibits a self-powered characteristic behavior, as shown in Figure 4c. In the self-supply voltage-detection mode, the device achieves a high responsivity of up to 1.1 mA/W. A large number of electron-hole pairs are generated in the film due to the internal electric field at the surface. The space charges separate and drift in opposite directions, generating photocurrent at the electrode to produce zero bias.

**Figure 5:**
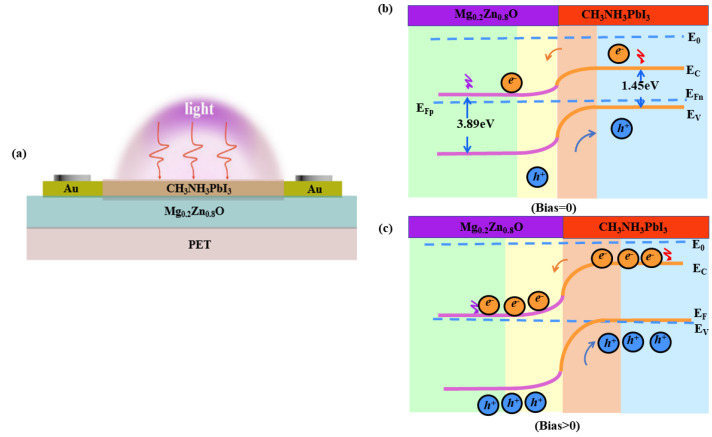
Diagram of the carrier transport mechanism. (**a**) Diagram of the carrier transport mechanism of the CH_3_NH_3_PbI_3_/Au/Mg_0.2_Zn0_.8_O SPPDs at 0 V. (**b**,**c**) Diagram of the carrier transport mechanism of the CH_3_NH_3_PbI_3_/Au/Mg_0.2_Zn0_.8_O heterojunction SPPDs under 0 bias and reverse bias.

Figure 5 shows a schematic of the carrier transport mechanism of the CH_3_NH_3_PbI_3_/Au/Mg_0.2_Zn_0.8_O heterojunction SPPDs. Such a mechanism can be explained by the band diagram. As can be seen from the figure, the band gaps of CH_3_NH_3_PbI_3_ and Mg_0.2_Zn_0.8_O are 1.45 and 3.89 eV, respectively. When the Mg_0.2_Zn_0.8_O film forms a heterojunction with the CH_3_NH_3_PbI_3_ film, the electrons in the former diffuse into the latter. Furthermore, the pores in the CH_3_NH_3_PbI_3_ film diffuse into the Mg_0.2_Zn_0.8_O film. When they are in equilibrium, a self-built electric field is formed at the heterojunction interface, as shown in Figure 5b. Under illumination, Mg_0.2_Zn_0.8_O absorbs UV light to produce photogenerated electrons and holes, while the CH_3_NH_3_PbI_3_ film absorbs UV, Vis, and NIR light to produce electrons and holes. Under the action of this self-established electric field, the photogenerated electrons and holes, which are diffused from the dissipative region of the heterojunction, rapidly separate; the electrons drift to the Mg_0.2_Zn_0.8_O film, and the holes drift to the CH_3_NH_3_PbI_3_ film; thus, a stable external current is generated. Therefore, self-driven CH_3_NH_3_PbI_3_/Au/Mg_0.2_Zn_0.8_O devices have a low dark current, a high light current, and high responsiveness. Due to the high recombination probability of the photogenerated electrons and holes in CH_3_NH_3_PbI_3_, the photocurrent enhancement of the CH_3_NH_3_PbI_3_/Au PDs is not very significant. However, when the n-Mg_0.2_Zn_0.8_O layer and the p-CH_3_NH_3_PbI_3_ perovskite material are introduced, a p-n junction with a well-matched band structure is formed, from p-CH_3_NH_3_PbI_3_ with a high Fermi level to n-Mg_0.2_Zn_0.8_O with a low Fermi level. The Fermi level of p-CH_3_NH_3_PbI_3_ in the p region gradually increases, while that of Mg_0.2_Zn_0.8_O in the n region gradually decreases, thus increasing the Fermi level [37]. Therefore, when the recombination probability of photogenerated electron-hole pairs in the CH_3_NH_3_PbI_3_ layer decreases, the photocurrent of the CH_3_NH_3_PbI_3_/Au/Mg_0.2_Zn_0.8_O heterojunction SPPDs increases significantly [38,39,40,41,42]. Thus, lowering the contact barrier on the surface results in a high photocurrent in the device. Therefore, the heterostructure PD can not only reduce the recombination probability of electrons and holes but also increase the depletion layer width, as shown in Figure 5c; the dark current decreases, so that the CH_3_NH_3_PbI_3_/Au/Mg_0.2_Zn_0.8_O heterojunction SPPDs have a high optical gain.

D* is a measurement of the detector’s sensitivity. Assuming that shot noise from the dark current is the major contributor to the total noise, it can be written as [43,44]:

(2) $$D*=\frac{R}{{\left(\frac{2qI_{d}}{A}\right)}^{\frac{1}{2}}}$$

where *R* is the responsivity, *A* is the area of the detectors, q is the unit charge, and *I_d_* is the dark current.

Due to the suppressed dark current and the enhanced responsivity of the CH_3_NH_3_PbI_3_/Au/Mg_0.2_Zn_0.8_O heterojunction SPPDs, as shown in Figure 4d, the best value of D* is as high as 4.7 × 10^12^ Jones at 780 nm, while it is only 3.0 × 10^10^ Jones in CH_3_NH_3_PbI_3_/Au devices. Notably, the CH_3_NH_3_PbI_3_/Au/Mg_0.2_Zn_0.8_O heterojunction PDs exhibit a pronounced response in the vis-light range as well as in the UV- and NIR-light ranges. It is found that the detectivity of the prepared perovskite SPPDs is greatly improved in the range of 300–800 nm, and a detectivity exceeding 2.34 × 10^12^ Jones is achieved in most of the range (320–780 nm). These results are comparable with those of other reports, and a detailed comparison is provided in Table 1. The table shows that the detection rate of the device prepared in this paper is higher than that of other devices, which have a detection rate of 4.7 × 10^12^ Jones and obtain ultra-low magnitude-dark currents compared to other devices, which have a dark current of 1.4 × 10^−1^ pA. The significant increase in detectivity indicates that the modification on both sides of the active layer results in a reduced trap density as well as improved carrier transport and extraction.

## 4. Conclusions

In summary, a CH_3_NH_3_PbI_3_ film was prepared and combined with an Mg_0.2_Zn_0.8_O film fabricated via vacuum magnetron sputtering to realize CH_3_NH_3_PbI_3_/Au/Mg_0.2_Zn_0.8_O heterojunction SPPDs. The results show that due to the addition of the Mg_0.2_Zn_0.8_O layer, the recombination probability of the photogenerated electron-hole pairs is reduced, and the optical gain is enhanced. Compared with the CH_3_NH_3_PbI_3_/Au PDs, the photoresponsivity is increased by nearly 53.17 times across the entire spectral range, and the EQE of the CH_3_NH_3_PbI_3_/Au/Mg_0.2_Zn_0.8_O heterojunction SPPDs is increased from 12.45% to 120%. Remarkably, in the self-supply voltage-detection mode, the SPPDs achieve a high responsivity of up to 1.1 mA/W. The dark current of the CH_3_NH_3_PbI_3_/Au/Mg_0.2_Zn_0.8_O heterojunction SPPDs is less than 1.4 × 10^−1^ pA at 0 V. The best value of the detectivity is as high as 4.7 × 10^12^ Jones.

## Figures and Tables

**Table 1:** The performance parameters of perovskite PDs in this and previously reported work.

Device Structure	*R* (A/W)	*I_dark_*	*EQE*	D*	*R*_0_ (mA/W)	Ref
CH_3_NH_3_PbI_3_/Au/Mg_0.2_Zn_0.8_O	0.58	1.4 × 10^−1^ pA	120	4.7 × 10^12^	1.1	This work
Ag/NP_3_/MAPbI_3_/Al	0.25	0.3 uA	-	1.53 × 10^11^	-	[5]
Al/Si/SiO_2_/MAPbI_3_/Pt	-	50 pA	-	8.8 × 10^10^	-	[6]
ZnO/CsPbBr_3_	0.01	-	-	-	-	[16]
Au/MoO_3_/MAPbI_3_/ZnO/FTO	0.05	1 nA	-	4.5 × 10^11^	-	[19]

## Data Availability

The data that support the findings of this study are available from the corresponding author upon reasonable request.

## References

[1] Khan M.F., Rehman S., Akhtar I., Aftab S., Ajma H.M.S., Khan K., Deok K.K., Eom J. (2020). High mobility ReSe_2_ field effect transistors: Schottky-barrier-height dependent photoresponsivity and broadband light detection with Co decoration. 2D Mater..

[2] Wei Y.C., Chen C., Tan C., He L., Ren Z.Z., Zhang C.Y., Peng S.L., Zhang C.Y., Han J.Y., Zhou H.X. (2022). High-Performance Visible to Near-Infrared Broadband Bi_2_O_2_Se Nanoribbon Photodetectors. Adv. Opt. Mater..

[3] Chen J.S., Li Y., Zhang Z.Q., Lu H. (2022). Structure design and properties investigation of Bi_2_O_2_Se/graphene van der Waals heterojunction from first-principles study. Surf. Interfaces.

[4] Kong L., Sun H.T., Nie Y.H., Yan Y., Wang R.Z., Ding Q., Zhang S., Yu H., He J., Luan G.Y. (2023). Luminescent Properties and Charge Compensator Effects of SrMo_0.5_W_0.5_O_4_: Eu^3+^ for White Light LEDs. Molecules.

[5] Liu B.W., Peng Y., Jin Z.M., Wu X., Gu H.Y., Wei D.S., Zhu Y.M., Zhuang S.L. (2023). Terahertz ultrasensitive biosensor based on wide-area and intense light-matter interaction supported by QBIC. Chem. Eng. J..

[6] Cai L.Y., LU Y.G., Zhu H.H. (2023). Performance enhancement of on-chip optical switch and memory using Ge_2_Sb_2_Te_5_ slot-assisted microring resonator. Opt. Laser Eng..

[7] Leung S.F., Ho K.T., Kung P.K., Hsiao V.K., Alshareef H.N., Wang Z.L., He J.H. (2018). A self-powered and flexible organometallic halide perovskite photodetector with very high detectivity. Adv. Mater..

[8] Li Y., Li Y., Zhou X. (2022). Flexible Optoelectronic Devices Based on Hybrid Perovskites. Mater. Devices.

[9] Bao C., Zhu W., Yang J., Li F., Gu S., Wang Y., Yu T., Zhu J., Zhou Y., Zou Z. (2016). Highly flexible self-powered organolead trihalide perovskite photodetectors with gold nanowire networks as transparent electrodes. ACS Appl. Mater. Interfaces.

[10] Sun H., Tian W., Cao F., Xiong J., Li L. (2018). Ultrahigh-performance self-powered flexible double-twisted fibrous broadband perovskite photodetector. Adv. Mater..

[11] Zhu Y., Song Z., Zhou H., Wu D., Lu R., Wang R., Wang H. (2018). Self-powered, broadband perovskite photodetector based on ZnO microspheres as scaffold layer. Appl. Surf. Sci..

[12] Pang T., Jia R., Wang Y., Sun K., Hu Z., Zhu Y., Luan S., Zhang Y. (2019). Self-powered behavior based on the light-induced self-poling effect in perovskite-based transport layer-free photodetectors. J. Mater. Chem. C.

[13] Chen Y., Chen T., Dai L. (2015). Layer-by-layer growth of CH_3_NH_3_PbI_3 − x_Cl_x_ for highly efficient planar heterojunction perovskite solar cells. Adv. Mater..

[14] Tong S., Wu H., Zhang C., Li S., Wang C., Shen J., Xiao S., He J., Yang J., Sun J. (2017). Large-area and high-performance CH_3_NH_3_PbI_3_ perovskite photodetectors fabricated via doctor blading in ambient condition. Org. Electron..

[15] Hu X., Zhang X., Liang L., Bao J., Li S., Yang W., Xie Y. (2014). High-performance flexible broadband photodetector based on organolead halide perovskite. Adv. Funct. Mater..

[16] Watanabe Y. (1995). Epitaxial all-perovskite ferroelectric field effect transistor with a memory retention. Appl. Phys. Lett..

[17] Xia H.-R., Li J., Sun W.-T., Peng L.-M. (2014). Organohalide lead perovskite based photodetectors with much enhanced performance. Chem. Commun..

[18] Zhang Y., Du J., Wu X., Zhang G., Chu Y., Liu D., Zhao Y., Liang Z., Huang J. (2015). Ultrasensitive photodetectors based on island-structured CH_3_NH_3_PbI_3_ thin films. ACS Appl. Mater. Interfaces.

[19] Hu Q., Wu H., Sun J., Yan D., Gao Y., Yang J. (2016). Large-area perovskite nanowire arrays fabricated by large-scale roll-to-roll micro-gravure printing and doctor blading. Nanoscale.

[20] Park N., Kang H., Park J., Lee Y., Yun Y., Lee J.-H., Lee S.-G., Lee Y.H., Suh D. (2015). Ferroelectric single-crystal gated graphene/hexagonal-BN/ferroelectric field-effect transistor. ACS Nano.

[21] Zhuo S., Zhang J., Shi Y., Huang Y., Zhang B. (2015). Self-template-directed synthesis of porous perovskite nanowires at room temperature for high-performance visible-light photodetectors. Angew. Chem..

[22] Liu D., Kelly T.L. (2014). Perovskite solar cells with a planar heterojunction structure prepared using room-temperature solution processing techniques. Nat. Photonics.

[23] Kim J., Kim G., Kim T.K., Kwon S., Back H., Lee J., Lee S.H., Kang H., Lee K. (2014). Efficient planar-heterojunction perovskite solar cells achieved via interfacial modification of a solgel ZnO electron collection layer. J. Mater. Chem. A.

[24] Dong X., Hu H., Lin B., Ding J., Yuan N. (2014). The effect of ALD-ZnO layers on the formation of CH_3_NH_3_PbI_3_ with different perovskite precursors and sintering temperatures. Chem. Commun..

[25] Bai F., Qi J., Li F., Fang Y., Han W., Wu H., Zhang Y. (2018). A high-performance self-powered photodetector based on monolayer MoS_2_/Perovskite heterostructures. Adv. Mater. Interfaces.

[26] Harrison S.E. (1954). Conductivity and Hall effect of ZnO at low temperatures. Phys. Rev..

[27] Hutson A.R. (1957). Hall effect studies of doped zinc oxide single crystals. Phys. Rev..

[28] Thomas D. (1957). Interstitial zinc in zinc oxide. J. Phys. Chem. Solids.

[29] Ghosh J., Natu G., Giri P. (2019). Plasmonic hole-transport-layer enabled self-powered hybrid perovskite photodetector using a modified perovskite deposition method in ambient air. Org. Electron..

[30] Li Y., Zhang Y., Li T., Li M., Chen Z., Li Q., Zhao H., Sheng Q., Shi W., Yao J. (2020). Ultrabroadband, ultraviolet to terahertz, and high sensitivity CH_3_NH_3_PbI_3_ perovskite photodetectors. Nano Lett..

[31] Su H., Meng L., Liu Y., Zhang Y., Hu M., Yang Z., Liu S.F. (2019). Effective electron extraction from active layer for enhanced photodetection of photoconductive type detector with structure of Au/CH_3_NH_3_PbI_3_/Au. Org. Electron..

[32] Gao L., Zeng K., Guo J., Ge C., Du J., Zhao Y., Chen C., Deng H., He Y., Song H. (2016). Passivated single-crystalline CH_3_NH_3_PbI_3_ nanowire photodetector with high detectivity and polarization sensitivity. Nano Lett..

[33] Chen Q., Zhou H., Hong Z., Luo S., Duan H.-S., Wang H.-H., Liu Y., Li G., Yang Y. (2014). Planar heterojunction perovskite solar cells via vapor-assisted solution process. J. Am. Chem. Soc..

[34] Duan Y., Zhang S., Cong M., Jiang D., Liang Q., Zhao X. (2020). Performance modulation of a MgZnO/ZnO heterojunction flexible UV photodetector by the piezophototronic effect. J. Mater. Chem. C.

[35] Willander M., Yang L.L., Wadeasa A., Ali S.U., Asif M.H., Zhao Q.X., Nur O. (2009). Zinc oxide nanowires: Controlled low temperature growth and some electrochemical and optical nano-devices. J. Mater. Chem..

[36] Youngblood N., Chen C., Koester S.J., Li M. (2015). Waveguide-integrated black phosphorus photodetector with high responsivity and low dark current. Nat. Photon..

[37] Aharon S., Gamliel S., El Cohen B., Etgar L. (2014). Depletion region effect of highly efficient hole conductor free CH_3_NH_3_PbI_3_ perovskite solar cells. Phys. Chem. Chem. Phys..

[38] Jošt M., Kegelmann L., Korte L., Albrecht S. (2020). Monolithic perovskite tandem solar cells: A review of the present status and advanced characterization methods toward 30% efficiency. Adv. Energy Mater..

[39] Liu P., Han N., Wang W., Ran R., Zhou W., Shao Z. (2021). High-quality ruddlesden–popper perovskite film formation for high-performance perovskite solar cells. Adv. Mater..

[40] Dai X., Xu K., Wei F. (2020). Recent progress in perovskite solar cells: The perovskite layer. Beilstein J. Nanotech..

[41] Hu Y., Schlipf J., Wussler M., Petrus M.L., Jaegermann W., Bein T., Muüller-Buschbaum P., Docampo P. (2016). Hybrid perovskite/perovskite heterojunction solar cells. ACS Nano.

[42] Zhang T., Long M., Qin M., Lu X., Chen S., Xie F., Gong L., Chen J., Chu M., Miao Q. (2018). Stable and efficient 3D-2D perovskite-perovskite planar heterojunction solar cell without organic hole transport layer. Joule.

[43] Ma C., Shi Y., Hu W., Chiu M.H., Liu Z., Bera A., Li F., Wang H., Li L.J., Wu T. (2016). Heterostructured WS_2_/CH_3_NH_3_PbI_3_ photoconductors with suppressed dark current and enhanced photodetectivity. Adv. Mater..

[44] Yu J., Chen X., Wang Y., Zhou H., Xue M., Xu Y., Li Z., Ye C., Zhang J., Van Aken P.A. (2016). A high-performance self-powered broadband photodetector based on a CH_3_NH_3_PbI_3_ perovskite/ZnO nanorod array heterostructure. J. Mater. Chem. C.

